# Dynamic Human Body Modeling Using a Single RGB Camera

**DOI:** 10.3390/s16030402

**Published:** 2016-03-18

**Authors:** Haiyu Zhu, Yao Yu, Yu Zhou, Sidan Du

**Affiliations:** School of Electronic Science and Engineering, Nanjing University, Nanjing 210023, China; MF1423044@smail.nju.edu.cn (H.Z.); coff128@nju.edu.cn (S.D.)

**Keywords:** SCAPE, non-rigid reconstruction, single RGB camera, pose estimation, motion classification, structure from motion

## Abstract

In this paper, we present a novel automatic pipeline to build personalized parametric models of dynamic people using a single RGB camera. Compared to previous approaches that use monocular RGB images, our system can model a 3D human body automatically and incrementally, taking advantage of human motion. Based on coarse 2D and 3D poses estimated from image sequences, we first perform a kinematic classification of human body parts to refine the poses and obtain reconstructed body parts. Next, a personalized parametric human model is generated by driving a general template to fit the body parts and calculating the non-rigid deformation. Experimental results show that our shape estimation method achieves comparable accuracy with reconstructed models using depth cameras, yet requires neither user interaction nor any dedicated devices, leading to the feasibility of using this method on widely available smart phones.

## 1. Introduction

Human body modeling has vast applications, such as computer games, animations and virtual fittings, and has long been an active topic in many research communities, from visual sensing and computer vision to computer graphics. Researchers have developed a number of approaches using depth images [1,2,3,4,5], 3D scanners [6,7] and multi-view images [8,9,10,11,12] to extract 3D human models over the last few decades. These approaches have progressively enabled faster and better production of 3D human models.

### 1.1. Human Body Modeling Using Depth Sensors

The emergence of depth sensors, such as the Kinect sensor, in recent years has made it possible to perform 3D reconstruction from depth or RGB-depth (RGB-D) images. Newcombe *et al.* [3] created a system called KinectFusion, which provides a detailed 3D reconstruction of fixed scenes, but this method can be applied only to static objects. Follow-up studies based on KinectFusion specifically focused on scanning humans, where a user rotates in front of the Kinect while maintaining a roughly rigid pose [4,13,14,15]. Zhang *et al.* [5] registered several Kinect scans of an object in multiple poses and used these data to train a personalized body model, which was then fitted to dynamic data. Newcombe *et al.* [2] extended the KinectFusion method to capture dynamic 3D shapes that included partial views of moving people by utilizing a dense volumetric warp-field parametrization. Bogo *et al.* [16] introduced a multi-resolution body model called Delta, which used a set of variable details for the shapes and locations of body parts along with a displacement map to capture shape details from images. Dou *et al.* [1] proposed a system that allowed a considerable amount of non-rigid deformation during scanning and achieved high quality results without heavily constraining users or camera motion by performing a dense non-rigid bundle adjustment to optimize the final shape and non-rigid parameters for each frame.

### 1.2. Multi-View Human Body Modeling

Multi-view stereo modeling uses multiple, sparsely-deployed cameras to observe a scene from different viewpoints. Gall *et al.* [8] recovered not only the movements of skeletons, but also the possible non-rigid temporal deformations from a multi-view image sequence using an unsupervised method that tracked the skeletons and consistently estimated the surface variations over time. Liu *et al.* [10] split the tracking problem into a multi-view 2D segmentation problem in which the segmentation separates persons by labeling each foreground pixel and a 3D pose and shape estimation problem. Huang *et al.* [9] introduced the notion of keyframes to 3D human motion tracking and proposed a keyframe-based tracking framework that updates the keyframe pool incrementally. In addition, they presented a new outlier rejection method to improve the method’s robustness and significantly limits the impact of missing data and outliers. Robertini *et al.* [11] proposed a new shape representation that models a mesh surface using a dense collection of 3D Gaussian functions centered at each vertex and formulated a dense photo-consistency-based surface refinement as a global optimization problem for the position of each vertex on the surface. Zollhofer *et al.* [12] utilized the volumetric fusion framework to create a multi-resolution hierarchy using a stereo camera and then employed real-time, non-rigid reconstruction to produce a deformed mesh at every time step.

### 1.3. Single-View Human Body Modeling

Guan *et al.* [17] fit a static model of human body poses and shapes to a single image using cues, such as silhouettes, edges and smooth shading. Hasler *et al.* [18] presented an approach to estimate a human body pose and shape from a single image using a bilinear statistical model. Although having several images of one subject can improve shape estimation, the human model still suffers from surface contortions. Jain *et al.* [19] re-projected a parametric 3D model onto an actor’s silhouette to optimize both the shape and pose parameters with the help of a Kanade-Lucas-Tomasi (KLT) tracker along with some manually-introduced features.

Unlike specialized setups or dedicated equipment for 3D reconstruction, using a single RGB camera, such as the integrated camera on mobile phones, has much more potential value for real-life applications. Although considerable attention has been paid to reconstructing human models from images or videos, e.g., acquiring shapes from shading [17], from silhouettes [9,10,17] and from model-based methods [18,19,20], some of these techniques are limited by assumptions concerning light source directions or intensities, while others may suffer from the insufficient constraint of single-image silhouettes or rely heavily on a good parametric model as the input. Zhou *et al.* [20] devised a method to compute shape completion and animation for people (SCAPE) parameters to match input images and enable a model-based morphing process for image editing. However, in this approach, users are required to carefully label the body parts and joints manually before computation, to prevent the method from getting stuck in local minima. Yu *et al.* [21] computed a dense 3D template of the object in a static pose from a short fixed sequence, which they used to subsequently perform non-rigid reconstruction.

Due to the high dynamics of the human body and the ambiguity that results from perspective projection, automatic human modeling using monocular RGB images or videos is still a challenging problem.

**Figure 1:**
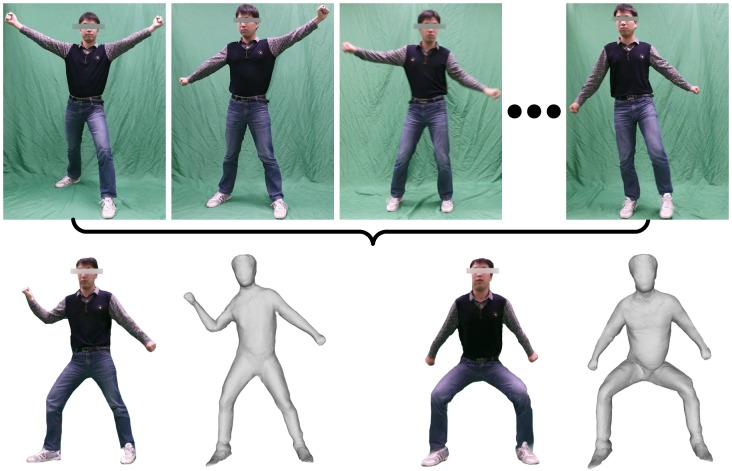
A performer is acting freely in front of a single camera, and our system automatically reconstructs a model of the performer. The first row shows the motion of the performer recorded by a common camera, and the second row shows 3D models that are obtained through our system and fitted to the poses in some of the frames from the video sequence.

To overcome the limitations described earlier, this paper proposes an automatic system that can model dynamic human bodies using a single RGB camera, as demonstrated in Figure 1. Compared to previous works, whose performance degrades when humans are in motion, we investigate whether human motion can be advantageous for improving human model reconstruction quality. The underlying feasibility of this approach arises from the fact that an object in motion may provide more cues for shape recovery than a static object, because self-occluded parts have a higher chance of being captured during motion. On the other hand, motion can be considered as an effective clue for human body segmentation, because coincident motion is more reliable evidence than texture, silhouette or location. To achieve this, we begin by designing a kinematic classification to relax the non-rigid recovery problem into a task of piecewise rigid reconstruction. The model for each rigid part is maintained and continuously improved as the image sequence acquisition process progresses. For each update of each part model, we initiate a non-rigid fusion method to merge the parts into a complete human model; thus, the human model is continuously and incrementally being built and improved along with the part models.

To the best of our knowledge, our system is the first that can automatically reconstruct a model for dynamic people from image sequences captured by a single RGB camera.

Our paper is organized as follows: Section 2 introduces the proposed approach. Experimental results and the discussion are shown in Section 3. Finally, we conclude our study in Section 4.

## 2. Building 3D Models

**Figure 2:**
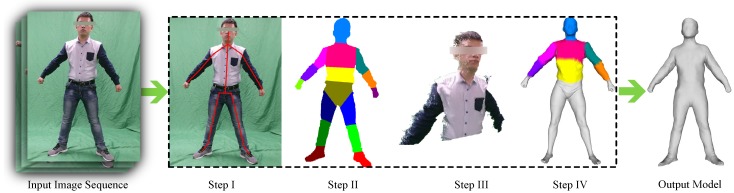
Our system’s pipeline begins by taking an image sequence as input, and its output is the reconstructed human model.

In this section, we supply the details of the pipeline depicted in Figure 2, which consists of the following steps: (i) Rather than manual labeling, we roughly derive 2D and 3D joint locations for each frame by running a software detector; (ii) The obtained 3D pose is refined via a carefully-designed kinematic classifier, and the body image is eventually segmented into rigid parts to relax the non-rigid problem into a rigid reconstruction problem; (iii) We propose a bundle adjustment-based approach to reconstruct the shapes of rigid parts by making full use of their motion flow. The reconstructed shape is continuously improved as more images are processed; (iv) A general template is finally deformed to fit the body parts, and the non-rigid component of shape deformation is derived to form a vivid 3D human model. As the rigid parts are continuously and incrementally being improved, the final shape of the human is also enhanced, along with the non-rigid deformation in each frame.

### 2.1. Initial Pose Detection

**Figure 3:**
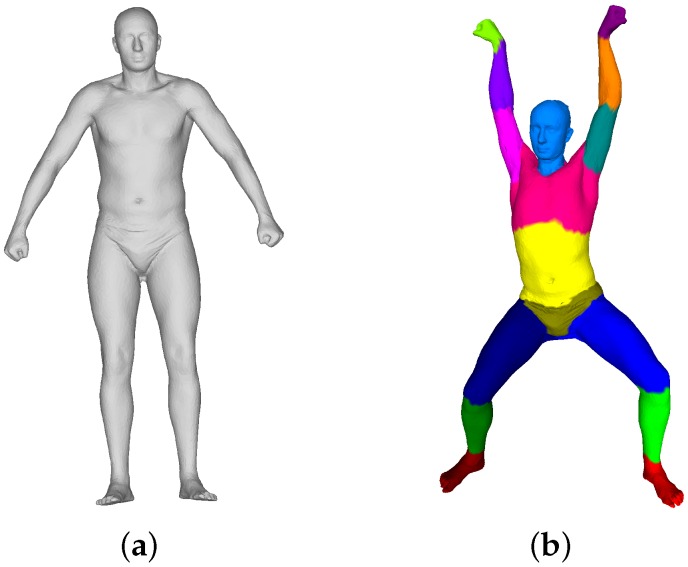
(**a**) Our template and (**b**) the template in a different pose with 16 color-coded parts.

In our framework, we adopt a general SCAPE model [6], illustrated in Figure 3, trained from the database provided by [22], to solve the pose transformation and non-rigid mesh deformation problems.

To deform the SCAPE model to the poses of the people in every frame, we need 3D poses of people to derive the pose transformations. To do this, we run a 2D pose detector [23] and a 3D pose parser [24] to roughly obtain the 3D poses for the given images in a frame-by-frame manner. Then, we refine each detected pose using a Kalman filter [25] to make it robust to noise and inaccurate 2D joint estimations. Specifically, we consider pose estimation as a linear dynamic system discretized in the time domain. In Equation (1), we can assume that $P_{i}$ (*i.e.*, the pose in the *i*-th frame) has evolved from $P_{i-1}$, because 3D poses of adjacent frames are highly related to each other, as follows:

(1) $$P_{i}=F_{i}P_{i-1}+B_{i}u_{i}+w_{i}$$

where $F_{i}$ is the state-transition model applied to the previous pose $P_{i-1}$ and $B_{i}$ is the control-input model for control vector $u_{i}$. The process noise, $w_{i}$, is assumed to have a zero-mean multivariate normal distribution with constant covariance. In the *i*-th frame, we obtain an estimation $Z_{i}$ of the true pose $P_{i}$ that satisfies Equation (2):

(2) $$Z_{i}=H_{i}P_{i}+v_{i}$$

where $H_{i}$ is the observation model and $v_{i}$ is the observation noise, which is assumed to be zero-mean Gaussian white noise with constant covariance [25]. After obtaining a pose from the pose estimator, we immediately refine it to be more reliable using the Kalman filter.

### 2.2. Pose Refinement via Kinematic Classification

The poses derived above implicitly provide a classification of rigid body parts for the given images; however, such a classification is usually far removed from the correct poses needed for further computation due to projective ambiguity and self-occlusion during movements. To achieve a better model, the initial roughly-detected poses are refined via a kinematic classifier. Note that non-rigid deformation is temporarily ignored here, but will be discussed later in Section 2.4. In physics, a rigid part is an ideal region that moves consistently; therefore, we can regard the regions in which points have similar motion as rigid body parts. We divide the body into 16 parts according to the skeletons and the SCAPE model.

**Figure 4:**
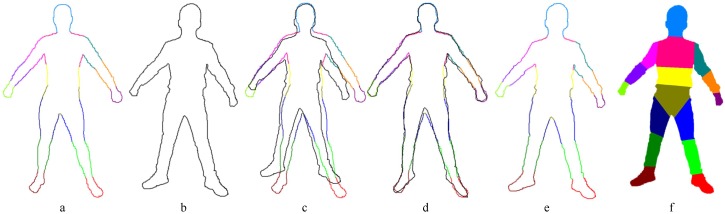
Stages in our kinematic classification. (**a**) The silhouette of our template projected to an image; (**b**) the silhouette of people in an image; (**c**) the result before alignment; (**d**) the aligned result after coherent point drift (CPD) [26]; (**e**) the classification of people’s silhouette obtained from our template; (**f**) the kinematic classification result. First, we extract the silhouettes from our template and people; then, we can classify the people’s silhouettes after aligning the silhouette in (**b**) to (**a**). Finally, we obtain the kinematic classifications (**f**) of the people in the images through a fully-connected conditional random field (CRF) model.

We model the image as a random field I defined over the variables $\left\{I_{1},\ldots ,I_{N}\right\}$, where $I_{i}$ is the color of pixel *i* at location $p_{i}$. A random field X is defined over a set of variables $\left\{X_{1},\ldots ,X_{N}\right\}$ to label each pixel *i* as $X_{i}$. We denote $d_{i}$ as the displacement of pixel *i*, which we obtain by the large displacement optical flow algorithm proposed by Brox and Malik [27]. The reduced Gibbs energy of the conditional random field is then calculated to produce a refined label $X_{i}$ for each pixel, as shown below:

(3) $$E\left(I,X\right)=\sum_{i}\psi_{u}\left(I_{i},X_{i}\right)+\sum_{i<j}\psi_{p}\left(X_{i},X_{j},I_{i},I_{j}\right)$$

where *i* and *j* range from one to *N*. The unary potential $\psi_{u}$ is evaluated according to how well the label *X* matches the input image *I*, derived as follows:

(4) $$\begin{array}{cc} & C\left(I_{i},X_{i}\right)=\underset{silhouette}{\underset{︸}{\omega_{s}u_{s}\left(p_{i},S\left(X_{i}\right)\right)}}+\underset{joints}{\underset{︸}{\omega_{j}u_{j}\left(p_{i},B\left(X_{i}\right)\right)}}+\underset{motion}{\underset{︸}{\omega_{m}u_{m}\left(d_{i},D\left(X_{i}\right)\right)}}\\ & s.t.\\ & \omega_{s}+\omega_{j}+\omega_{m}=1 \end{array}$$

where $\omega_{s}$, $\omega_{j}$ and $\omega_{m}$ are weighting factors and $S\left(X_{i}\right)$ returns the set of contour points for each labeled $X_{i}$. Such points are computed by projecting our template to the image. The template silhouette is aligned to the image silhouette via coherent point drift (CPD) [26]. An example of the process is illustrated in Figure 4. The silhouette energy $u_{s}$ is derived from the minimum square distance between the location $p_{i}$ and the contour points:

(5) $$u_{s}\left(p_{i},S\left(X_{i}\right)\right)=\exp\left(-\frac{\min\left({\left|p_{i}-S\left(X_{i}\right)\right|}^{2}\right)}{2\theta_{s}^{2}}\right)$$

$B\left(X_{i}\right)$ is the barycenter of the bone assigned to label $X_{i}$ in the image; thus, joint energy $u_{j}$ is calculated as follows:

(6) $$u_{j}\left(p_{i},B\left(X_{i}\right)\right)=\exp\left(-\frac{{\left|p_{i}-B\left(X_{i}\right)\right|}^{2}}{2\theta_{j}^{2}}\right)$$

where the parameters $\theta_{s}$ and $\theta_{j}$ control the connection strength and range of pixels.

To justify the classification, a motion energy term $u_{m}$ is proposed to judge how the motion of $I_{i}$ matches the motion of the rigid part $X_{i}$, where $D\left(X_{i}\right)$ is the displacement of the barycenter of the body part assigned to label $X_{i}$, and the motion energy is evaluated to determine how well the motion of $I_{i}$ agrees with the motion of the barycenter of the body part labeled by $X_{i}$:

(7) $$u_{m}\left(d_{i},D\left(X_{i}\right)\right)=\rho_{d}\left(1+\frac{d_{i}•D\left(X_{i}\right)}{\left|d_{i}\right|\left|D\left(X_{i}\right)\right|}\right)$$

The pairwise potentials $\psi_{p}$ in Equation (3) are obtained by:

(8) $$\begin{array}{cc} \psi_{p} & \left(X_{i},X_{j},I_{i},I_{j}\right)=\mu \left(x_{i},x_{j}\right)\exp\left(-\frac{{\left|p_{i}-p_{j}\right|}^{2}}{2\theta_{\alpha }^{2}}-\frac{{\left|X_{i}-X_{j}\right|}^{2}}{2\theta_{\beta }^{2}}\right)+\mu \left(x_{i},x_{j}\right)\exp\left(u_{m}\left(d_{i},d_{j}\right)-1\right) \end{array}$$

where $\theta_{\alpha }$ and $\theta_{\beta }$ are parameters to control the degrees of nearness and similarity. The label compatibility function $\mu \left(x_{i},x_{j}\right)$ is provided by the Potts model, $\mu \left(x_{i},x_{j}\right)=\left[x_{i}\neq x_{j}\right]$. As a result, $\psi_{p}$ tends to make pixels within an area with similar motion more likely to be labeled as the same part.

**Figure 5:**
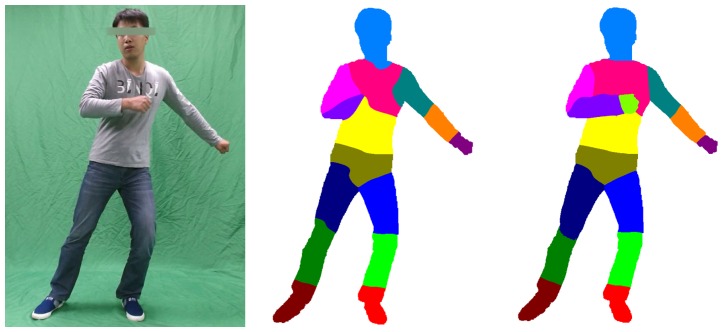
Body part kinematic classification. The left image is the source image recorded by an RGB camera, while the middle image is the classification result without the motion term. The classification result shown in the right-hand image is more precise than the result in the middle image, because of the motion term.

We use the mean field method with an efficient implementation proposed by Krahenbuhl *et al.* [28] to optimize Equation (3). To show the importance of the motion term in Equation (4), we assign $\omega_{m}=0$, and we solve the dense CRF model in the same way. Figure 5 demonstrates that the classification with the motion term (the right figure in Figure 5) is more precise than the classification without the motion term (the middle figure in Figure 5), which results in incorrect classifications, such as the right hand and the fuzzy classification of the right leg. However, with the help of the motion term, our kinematic classification not only provides a clear classification of the right leg, but also distinguishes between the right hand and the chest.

### 2.3. Dense Reconstruction

By performing kinematic classification in each frame of the sequence, we transform the non-rigid reconstruction into a rigid reconstruction that can be achieved by a structure from motion-based approach. In practice, to enable our system to tackle images with sparsely-detected features and unstable detection for shapes in non-rigid motion, we make use of the dense optical flow approach to perform reconstruction, instead of using sparse features, as demonstrated in Algorithm 1.

**Algorithm 1 Dense Reconstruction of Body Parts**1:*i*: the frame number of the input image2:$l_{i}^{b}$: the 2D barycenter of $B_{b}$ in the frame $f_{i}$3:$f_{b}$: the number of start frame of part $B_{b}$, denoted as ref for simplification4:**for**
b←1
**to** 16 **do**5:  **if**
$∥l_{i}^{b}-l_{ref}^{b}∥\;>$ 30 **then**6:      $T_{ref}^{b}\leftarrow T_{ref}^{{}^{\prime }}$7:      **for**
$j\leftarrow f_{b}+1$
**to**
*i*
**do**8:            $T_{j}^{b}\leftarrow \Delta T_{ref\to j}^{b}T_{ref}^{b}$9:            derive dense optical flow from $f_{ref}$ to $f_{j}$10:      **end**
**for**11:      $P^{b}\leftarrow \min E_{BA}$12:      $f_{b}\leftarrow f_{i}$13:  **end**
**if**14:**end for**

Here, $f_{i}$ represents the *i*-th frame in the image sequence, and $B_{i}$ represents the *i*-th body part of a person. For each part, we regard the motion of parts over a short duration as rigid motion and track the motion throughout the image sequence, by using the large displacement optical flow (LDOF) algorithm [27] to obtain a dense optical flow with sub-pixel accuracy. When the motion of any part, e.g., $B_{b}$ reaches a threshold value (20 pixels), we choose the frames from $f_{b}$ (in which the part starts to move) through $f_{i}$ (where the motion of this part reaches the threshold) to perform the bundle adjustment. The threshold is set according to the re-projection error obtained through bundle adjustment (Equation (9)). When the threshold is small, the depth of the points is highly uncertain because of the short baseline, which contributes to the large re-projection error. However, if we choose a large threshold, which means that more non-rigid components of the motion will be involved in the bundle adjustment, the re-projection error is also large. To set the threshold to an appropriate value, we increase it gradually, starting from a small value, and choose the value at which the re-projection error reaches a minimum. The start frame index $f_{b}$ is then updated to the current frame index $f_{i}$. Let *π* : ${ℜ}^{3}\to {ℜ}^{2}$ be the projection function; then, the cost function of the bundle adjustment is defined as:

(9) $$E_{BA}=\sum_{j=f_{b}}^{i}\sum_{k\in B_{b}}{∥p_{j}^{k}-\pi (r_{j}^{b}P_{k}+t_{j}^{b})∥}^{2}$$

where the 3D location of the *k*-th point in part $B_{b}$ is denoted as $P_{k}$, whose projection on the *j*-th frame is $p_{j}^{k}$, and $T_{j}^{b}=\left[r_{j}^{b}|t_{j}^{b}\right]$ is the relative camera pose viewing $B_{b}$ of the *j*-th frame. Note that the relative camera pose, denoted as $T_{j}^{b}$, is not the absolute camera pose $T_{j}^{*}$; the relative camera pose is used here to measure the relative motion between the camera and each part $B_{b}$ individually, including both the camera motion and the part motion. For instance, taking the motion of $B_{b}$ into consideration, we can obtain the matrix $\Delta T_{f_{b}\to j}^{b}$ of $B_{b}$, transforming from the reference frame to the *j*-th frame by driving the skeleton. Thus, $T_{j}^{b}=\Delta T_{f_{b}\to j}^{b}T_{f_{b}}^{*}$.

**Figure 6:**
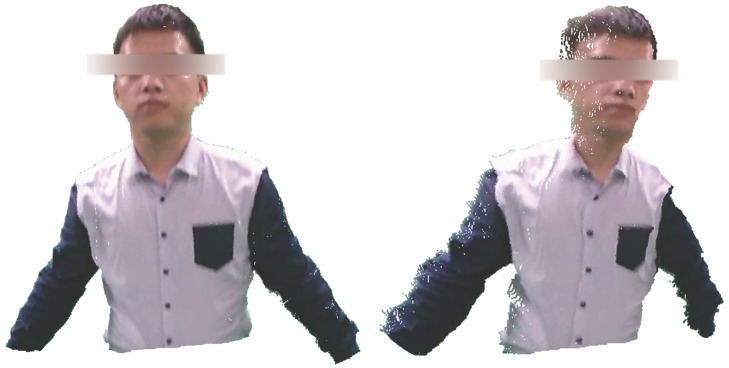
Dense reconstruction of seven body parts.

We utilize sparse bundle adjustment [29] to minimize the cost Equation (9), and all of the points are initially set to a random depth between two and four meters. Finally, we make use of the dense correspondences of pixels to reconstruct the 3D surfaces of parts. An example of dense reconstruction is shown in Figure 6.

### 2.4. Decomposing the Deformation

In this section, we decompose the deformation of the human shape into rigid and non-rigid components. The rigid component of motion consists of both the rigid motion of the body shape and that of clothing. To achieve this, we further process the reconstructed body parts, using the temporal information within each reconstructed part model, to compute the rigid component of the human model and the non-rigid component for the human deformations in each frame.

#### 2.4.1. Building Vertex-Wise Correspondence

**Figure 7:**
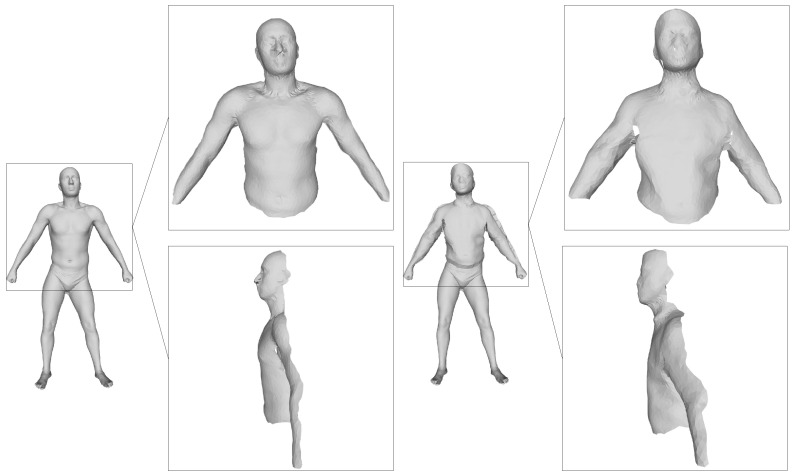
We obtain the dense reconstruction of upper parts of the body. The left figure is our template driven by the current reference frame before deformation. The right figure is an instance obtained through deforming several body parts of our template.

The rigid component of the human model is a pose-invariant deformation for a given performer in the image sequence. This means that such a component will implicitly form a coincident deformation along all of the frames. To investigate this pose-invariant deformation, a vertex-wise correspondence must be built. Unfortunately, the reconstructed shapes of body parts contain only the geometry information of the body parts in each frame and do not maintain semantic information for vertex-wise correspondence. Therefore, we use the general template used in the kinematic classification to merge the body part over time, by aligning the template and the recovered models of body parts. Note that the general template can look quite unlike the recovered people in Figure 3, *i.e.*, the difference between the template and the human model affects neither the aforementioned kinematic classification nor the merging process here. Specifically, we drive the template to fit the poses in the reference frames of the body part that are used in the dense reconstruction and vary during the process of reconstruction. Then, the visible points only are transformed to the dense reconstruction by the Z-buffer algorithm and aligned to the dense reconstruction through CPD. Eventually, an instance is obtained. A demonstration of the merging results is shown in Figure 7.

**Figure 8:**
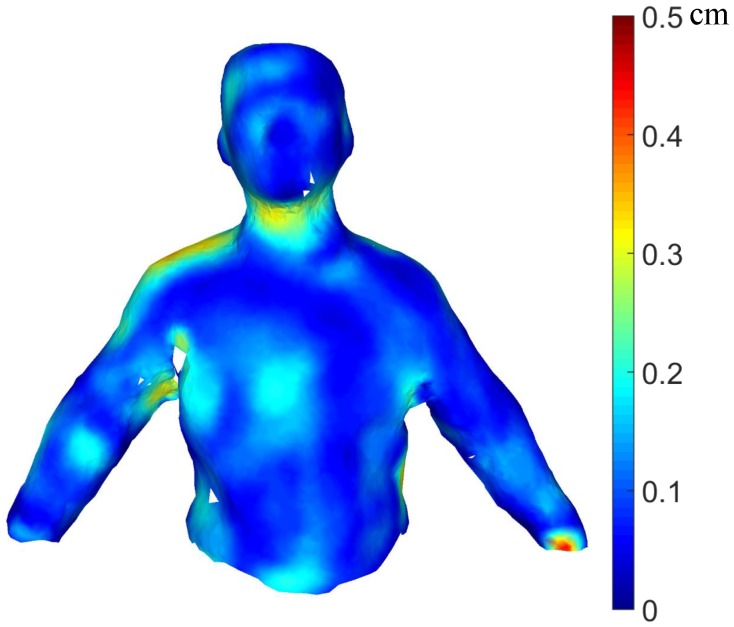
The error map of deforming some body parts of the template.

To validate the CPD algorithm in our point registration, we map the Euclidean distances of the correspondent points provided by CPD to the color space. The registration error is shown in Figure 8. As the preceding figure demonstrates, the registration result is acceptable. The maximum error is 0.5 cm, located in a small region of the left arm. The number of points transformed in the template is 2689, and the mean registration error is approximately 0.8 mm. Although the registration result contains some noise points, they have little effect on the following steps.

#### 2.4.2. Deriving Non-Rigid and Rigid Components

To decompose the rigid component, which is the pose-invariant deformation of a particular human shape, and the non-rigid component that arises from the pose in each frame, we combine all instances to derive deformation matrices. Our model deformations are calculated in a way similar to the SCAPE-based methods [6], where the obtained model consists of a generic template mesh model and a set of instance meshes. We use a 3×3 matrix $D_{k}^{j}$ to represent the deformation of each polygon, which is derived by solving the cost function below:

(10) $$\begin{array}{c} \min_{D^{j},R^{j}}\sum_{k}\sum_{l=2,3}\rho ∥R_{k}^{j}D_{k}^{j}{\hat{u}}_{k,l}-u_{k,l}^{j}{∥}^{2}+\rho \omega_{d}\sum_{k_{1},k_{2}}{∥D_{k_{1}}^{j}-D_{k_{2}}^{j}∥}^{2} \end{array}$$

where $R_{k}^{j}$ is a rigid rotation and ${\hat{u}}_{k,l}=v_{k,l}-u_{k,1},l=2,3$ are two edges of triangle *k* in our template mesh. Similarly, $u_{k,l}^{j}$ are two edges of triangle *k* in instance *j*. *ρ* is an indicator function. After the triangle *k* in instance *j* has been deformed, ρ=1; otherwise, ρ=0. $\omega_{d}=1e^{-3}$ is used to not only prevent large deformation changes among adjacent triangles, but also to reduce the negative influences caused by the noise points in dense reconstruction. Because the deformation matrix $D_{k}^{j}$ and rotation matrix $R_{k}^{j}$ are both unknown, the optimization is nonlinear. Therefore, we optimize the problem by making an initial guess for $R_{k}^{j}$ and then solve for $D_{k}^{j}$ and $R_{k}^{j}$ individually and iteratively. After $D_{k}^{j}$ is obtained, the rotation matrix $R_{k}^{j}$ can be updated again by a twist vector *ω*, $R_{k}^{j\;new}\leftarrow \left(I+{\left[\omega \right]}_{\times }\right)R_{k}^{j\;old}$, in which ${\left[\cdot \right]}_{\times }$ denotes the cross product matrix. Thus, when $D_{k}^{j}$ is solved, the twist vector *ω* is solved by minimizing:

(11) $$\begin{array}{c} \min_{\omega^{j}}\sum_{k}\sum_{l=2,3}\rho ∥\left(I+{\left[\omega \right]}_{\times }\right)R_{k}^{j}D_{k}^{j}{\hat{u}}_{k,l}-u_{k,l}^{j}{∥}^{2}+\omega_{t}\sum_{b1,b2}{∥\omega_{b_{1}}-\omega_{b2}∥}^{2} \end{array}$$

where $b_{1}$ and $b_{2}$ denote two neighboring body parts. After alternatively updating $D_{k}^{j}$ and $R_{k}^{j}$ until they converge to a local minima, a set of matrices $D^{j}$ is obtained.

Because our general template contains *K* points, the deformation matrices $D^{j}$ for each instance can be converted to a vector with size 9×K, which can be generated from a simple linear subspace: $D^{j}=U\beta^{j}+\mu$. Here, *μ* is the pose-invariant deformation of each person, which is obtained by calculating the mean value of $D^{j}$. When the number of instances is sufficient, we can derive the non-rigid component $\beta^{j}$ easily by principal component analysis (PCA).

## 3. Results and Discussion

**Figure 9:**
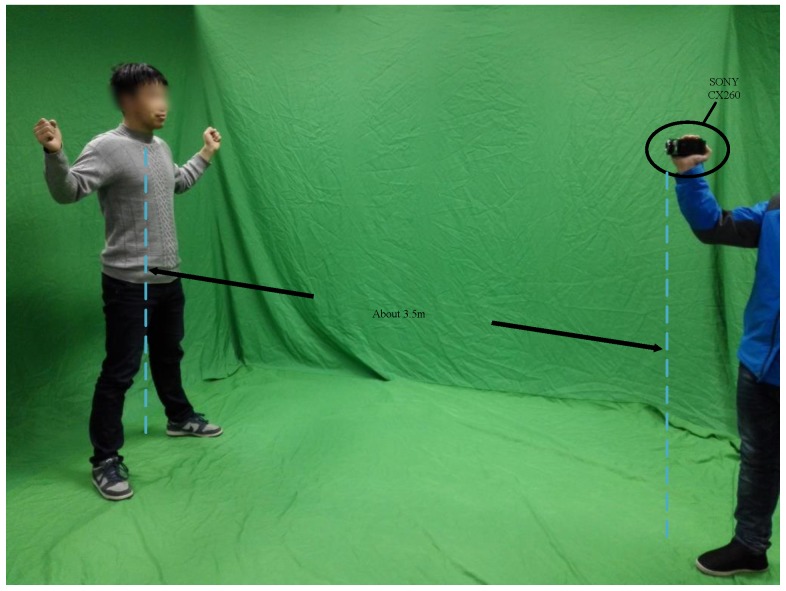
Our setup consists of a common camera, which can record the whole body of the people at an appropriate distance.

In the following experiments, we validate our method on a dataset containing a variety of RGB videos of 30 people performing various types of motion. Our system was developed with MATLAB and executed on an Intel i7 CPU at 3.40 GHz with 24 GB RAM. The images in the dataset were all recorded at a resolution of 1920×1080 using SONY CX260 under conditions illustrated by Figure 9 and processed off line. Each sequence consists of approximately 450 frames. On average, we obtain an instance from every ten frames. To evaluate the accuracy of our method, we also used KinectFusion and a non-rigid reconstruction algorithm to reconstruct 3D models of the same person in the RGB video for comparison. Furthermore, we also compared our method with the system introduced by Xu *et al.* [30], which can measure body parameters of dressed people with large-scale motion using a Kinect sensor.

### 3.1. Parameters

Some parameters not stated previously in this paper above were set to fixed values throughout our experiments. These parameter values are listed in Table 1.

To determine the values of the parameters in Table 1, we utilized a method similar to the method described in [28]. Although we need to set the values of six parameters listed in the first two rows in Table 1, $\rho_{d}$ is set to 0.5 to normalize the motion energy term in Equation (7); therefore, we only need to choose the appropriate values for $\omega_{s},\omega_{j},\theta_{s},\theta_{j}$ because of Equation (4). We manually labeled the different body parts in 20 images of the same person in different poses as the ground truth, then varied the values of $\omega_{s}$, $\omega_{j}$, $\theta_{s}$ and $\theta_{j}$ and, finally, analyzed the resulting classification accuracy. The parameters are set to the values that maximize the classification accuracy. The settings for the parameters $\theta_{\alpha }$ and $\theta_{\beta }$ were determined in the same manner.

### 3.2. Qualitative Analysis

**Figure 10:**
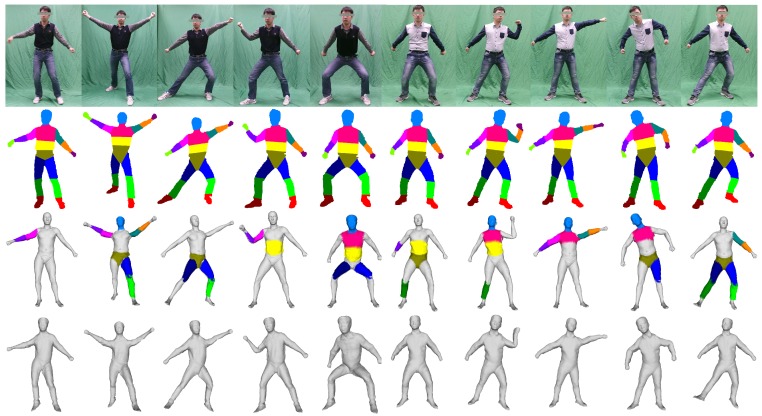
Some of the results. The first row shows some representative frames of our dataset. The second row shows the 2D kinematic classification. The third row shows the deformation of our template, the deformed parts are marked in different colors. The last row is the model driven by the current poses.

We show both the recovered poses and shapes across different bodies with various poses in the example, illustrated in Figure 10. Although we do not employ any optimization-based method for the pose parameters or during local searches in the pose space to improve the resolved poses, our method achieves a fairly accurate computational result for the given image sequence. The second row of Figure 10 demonstrates the effectiveness of kinematic classification. Our method requires no assumptions to reconstruct people. As depicted in the third row of Figure 10, the general template appears quite unlike the reconstructed people, *i.e.*, the proposed system can be utilized in a general application without making any previous body measurements. We maintain the human model and continuously improve the results as more images are processed. The parts of the body improving during the process are highlighted with different colors in the third row. At each processing step, the full body model is also improved, because the enhanced result for the individual parts also contributes more details to the overall model’s shape. Reconstructed models are driven individually to fit the poses in the images, to show the effectiveness of both the poses and the recovered shapes, as illustrated in the last row of the Figure 10.

**Figure 11:**
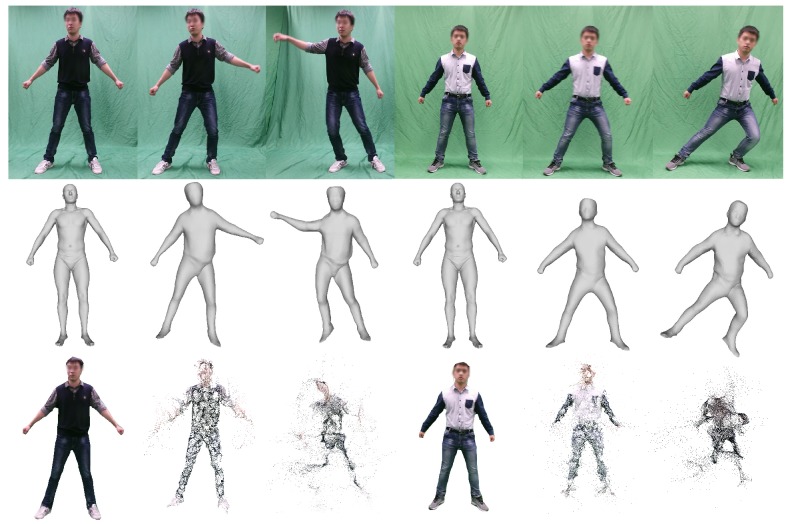
Results from two different motion sequences. The middle row is some of the results from our template corresponding to frames of the motion sequences shown in the first row. The last row shows the template required by Yu’s algorithm [21] and some of the results obtained through their system.

We make a comparison with a state of the art non-rigid reconstruction algorithm proposed by Yu *et al.* [21], which needs to compute a dense 3D template from a rigid sequence and then conducts the non-rigid deformation of a dynamic object. Obviously, their method requires more user interaction to ensure that the object to be reconstructed is static at the beginning of the frame sequence. The static object is required to compute a template for every scene. In contrast, our general SCAPE model can be applied to all scenes directly without requiring a rigid sequence. In addition, the examples illustrated in their paper are small objects in a closeup view, e.g., small bobby, toy pig, *etc.* We tested their algorithm on our dataset. The results are shown in Figure 11. Although two high quality templates are obtained through a dense reconstruction algorithm (see the first and fourth figure in the last row in Figure 11), the non-rigid reconstruction obtained is messy (see the last row of Figure 11), where an increasing number of noise points emerge for large performers’ movement. Because their model dose not include a skeleton-driven method, it is arduous for Yu’s system [21] to tackle the large motions of dynamic people. However, precisely because of the motion, our system polishes the reconstruction quality of two different people using the same template (see the first and fourth figure in the second row demonstrated in Figure 11). In addition, our SCAPE model can automatically make inferences about the body parts that are invisible in any single view; however, Yu’s results are limited to reconstructing only the visible parts of a human body. There is no doubt that our system is more effective at reconstructing 3D models of dynamic people through a single RGB camera, even when the view of the object is relatively distant.

### 3.3. Quantitative Analysis

**Figure 12:**
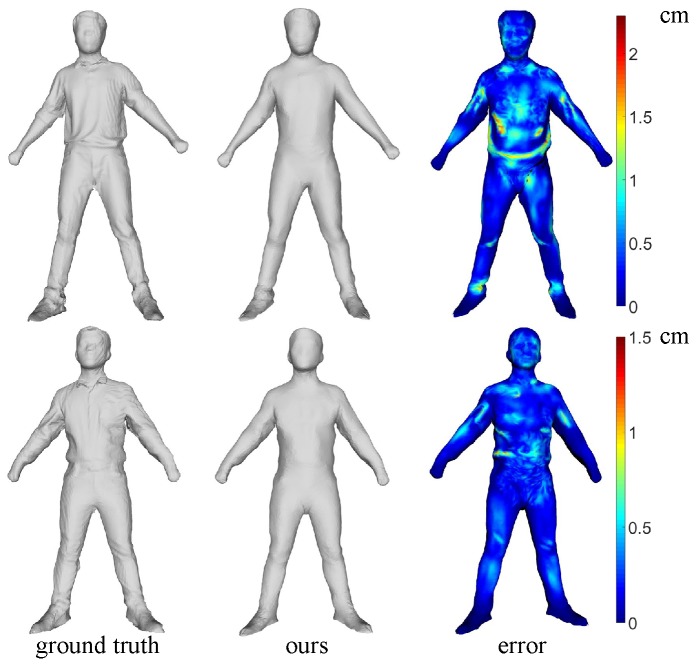
The watertight reconstruction of different people.

To our knowledge, no other method can automatically recover the shapes of dynamic people from a single RGB camera; we show only the quantitative comparison between our system and KinectFusion. The model is recovered using KinectFusion for people in a static pose. The template we use contains 12,500 points and 25,000 faces. To precisely measure our reconstruction error, we align the front part of our model to the KinectFusion results using the rigid iterative closest point (ICP) algorithm and map the Euclidean distance of the closest points to the color space to show the reconstruction error intuitively. As Figure 12 shows, the average reconstruction errors are 0.3 cm and 0.2 cm for the first sequence (the first row in Figure 12) and the second sequence (second row in Figure 12), respectively. The maximum reconstruction error is 1.5 cm for all sequences in our dataset. In the first sequence results, the error occurs mainly around the waist, where the clothing is very loose. There are some different poses in the second sequence (the first row in Figure 10). Note that the watertight model shown in the second row in Figure 12 has less reconstruction errors than the first one due to the tighter clothing, which shows the robustness of our system.

The final model derived from our system can be applied to many areas, particularly for virtual fittings. In our experiment, we reconstructed models for 30 different people and measured the body parameters following the method described by Xu [30]. The mean errors of body parameters are calculated and compared to Xu’s results in Table 2. Although Xu’s method utilizes a depth sensor to improve the precision of body parameter measurement, our system is superior to Xu’s in both convenience and precision: the mean errors of chest girth, neck to hip distance and thigh girth are smaller than Xu’s, even though our method uses only a single RGB camera.

**Figure 13:**
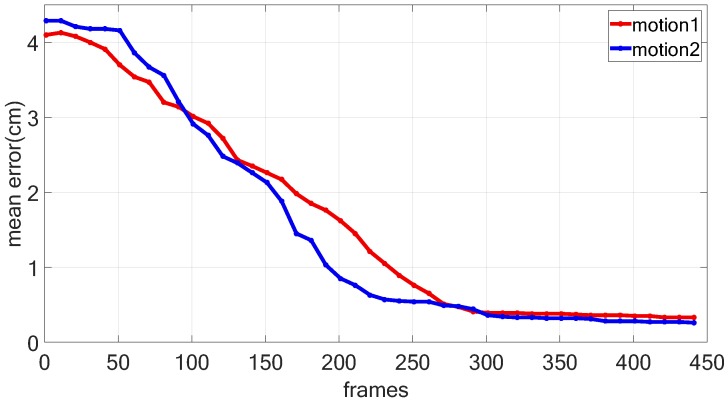
The average reconstruction error between the model and KinectFusion. The horizontal ordinate represents the number of frames processed. The vertical coordinate represents the average reconstruction error. The average reconstruction error decreases along with the increase of frames.

To test the influences of the number of frames on the quality of reconstructed models, we evaluate the relationship between the mean errors of reconstructed models and the number of frames in Figure 13. Experimental results show that we can obtain high quality reconstructed models when the input sequence contains more than 400 frames.

### 3.4. Computation

Due to the large amount of computation required for 3D pose estimation, the dense optical flow descriptor, dense CRF and bundle adjustment, our system is time consuming, currently requiring approximately 8 min per-frame, of which the kinematic classification costs approximately 5 min. In our experiments, we find that the most time-consuming steps are pose estimation and kinematic classification, which together consume approximately 90% of the execution time. The complexity of the pose estimation is O(N), where *N* is the image size. This complexity is analyzed clearly in [23]. According to the implementation of dense CRF detailed in [28], the main steps involved in solving the dense CRF problem consist of a message passing step, a compatibility transform and a local update. The computational bottleneck is message passing, which also has a complexity of O(N) with a high-dimensional filtering algorithm, where *N* is the image size. Therefore, our system has a total complexity of O(Nn), where *n* is the number of frames processed.

Still, even though our system is time consuming, it is the first method that can reconstruct a 3D model of a dynamic person from a single RGB camera. Moreover, our system can be parallelized; we will optimize the performance of our system in future work.

## 4. Conclusions

In this paper, we presented an automatic system to create dynamic human models from single-camera RGB videos. Our system first refines the coarse 2D and 3D poses using kinematic classification, which is aided by human body motion. From the dense reconstruction of the body parts, a personalized parametric model is constructed incrementally by calculating rigid and non-rigid deformation components. Using that personalized model, our system is ready for testing in some real-life applications, such as trying on and fitting virtually. It should be noted that our method requires no special user interactions or any dedicated devices. Moreover, it is particularly user friendly in that people can employ their existing devices (e.g., smart phones) to create accurate 3D models of the human body.

Regarding future works, we will primarily focus on extending our method to capture more finely detailed models and on improving the speed of our system.

## Figures and Tables

**Table 1:** The parameter settings for our experiments.

Equations		Values of Parameters	
Equation (4)	$\;\;\;\omega_{s}=5/16$	$\;\;\;\omega_{j}=1/16$	$\omega_{m}=5/8\;$
Equations (5–7)	$\theta_{s}=60$	$\theta_{j}=60$	$\rho_{d}=0.5$
Equation (8)	$\theta_{\alpha }=60$	$\theta_{\beta }=25$	

**Table 2:** Comparison of body parameters with Xu’s.

Errors	Arm Length	Chest Girth	Neck to Hip Distance	Hip Girth	Thigh Girth
Error of Xu’s (cm)	**1.2**	3.2	4.5	**3.1**	2.1
Error of Ours (cm)	1.4	**2.3**	**3.7**	3.3	**1.9**
